# Tics as an initial manifestation of juvenile Huntington’s disease: case report and literature review

**DOI:** 10.1186/s12883-017-0923-1

**Published:** 2017-08-08

**Authors:** Shi-Shuang Cui, Ru-Jing Ren, Ying Wang, Gang Wang, Sheng-Di Chen

**Affiliations:** Department of Neurology & Neuroscience Institute, Ruijin Hospital affiliated to Shanghai Jiao Tong University School of Medicine, Shanghai, 200025 China

**Keywords:** Juvenile Huntington’s disease, Tics, Case report, Literature review

## Abstract

**Background:**

Huntington’s disease (HD) is an autosomal dominant disorder, typically characterized by chorea due to a trinucleotide repeat expansion in the HTT gene, although the clinical manifestations of patients with juvenile HD (JHD) are atypical.

**Case presentation:**

A 17-year-old boy with initial presentation of tics attended our clinic and his DNA analysis demonstrated mutation in the HTT gene (49 CAG repeats). After treatment, his symptoms improved. Furthermore, we performed literature review through searching the databases and summarized clinical features in 33 JHD patients.

**Conclusion:**

The most prevalent symptoms are ataxia, and two cases reported that tics as initial and prominent manifestation in JHD. Among them, 88% patients carried CAG repeats beyond 60 and most of them have family history. This case here illustrates the variable range of clinical symptoms of JHD and the necessity of testing for the HD mutation in young patients with tics with symptoms unable to be explained by Tourette’s syndrome (TS).

## Background

Huntington’s disease (HD) is an autosomal dominant movement disorder, typically characterized by chorea, cognitive decline, and behavioral changes, due to a trinucleotide (CAG) repeat expansion in the HTT gene. Healthy individuals typically have fewer than 35 CAG repeats, and repeats of 40 or above cause HD with complete penetrance [1]. Except for Chorea, other symptoms include eye-movement abnormalities, parkinsonism, dystonia, myoclonus, tics, ataxia, dysarthria and dysphagia, and spasticity with hyperreflexia [1]. Its age at onset (AAO) ranges from 1.5 to 85 years [2]. Here we report a boy whose prominent symptoms were those typically associated with Tourette’s syndrome (TS) initiated at age of 9, confirmed HD with the presence of expanded CAG repeats.

## Case presentation

A 17-year-old boy first noted the gradual onset of prompt, repeated irregular tics of his head with uncontrolled eye blinking, frowning, and sometimes throat clearing 8 years ago. These involuntary movements had gradually involved in his shoulders, right upper limb and both lower limbs, and torticollis developed since 5 years earlier, exacerbated by anxiety and disappearing during sleep. The tics of right upper limb were worse with a heavy load and suppressed by himself occasionally. He complained about both inattention and hyperactivities in class when studying in school. He had been inclined to obsessive hand washing in the last 5 years, and had night terrors occasionally which could not be recalled anything after sleeping. He denied the presence of choreic movement, loss of consciousness, urinary or fecal incontinence and obvious cognitive decline. He had been to several hospitals without confirmed diagnosis and taken Chinese traditional medicine irregularly.

The patient was full-term. His birth and developmental milestones were normal and he denied any disease including diabetes mellitus and carpal tunnel syndrome. He is in high school and of mid-ranking. His younger brother is healthy and he had no family history of neurologic disorders including tics, HD, obsessive-compulsive disorder (OCD), attention deficit hyperactivity disorder (ADHD). Past medical history reveals no history of head injury, surgery and streptococcus infection. His parents also deny any symptoms of tics, HD, OCD and ADHD.

The most prominent finding of neurologic examination was the presence of multifocal tics manifested chiefly by blinking, frowning, head jerking, shoulder shrugging. Physical examination showed a spasmodic torticollis and hypertrophic left sternocleidomastoideus muscle. Neuropsychiatric assessment revealed the evidence of mild depression with Self-Rating Depression Scale score of 54 (of 80), mild anxiety with Self-Rating Anxiety Scale score of 56 (of 80). The Mini-Mental State score was 29/30 and Montreal Cognitive Assessment score was 30/30, suggested that no evidence of cognitive impairment. Both Kayser-fleischer ring and choreic movement were absent. Deep reflexes were brisk and Babinski’s sign was absent. Speech and muscle tone was normal.

Blood routine and smear, liver function, iron and copper metabolism were of normal range. Cranial Magnetic resonance imaging (MRI) including susceptibility weighted imaging (SWI) and diffusion weighted imaging (DWI) was unremarkable. Ambulatory electroencephalography (AEEG) implied paroxysmal epileptic discharges during sleep. Electromyography implied spontaneous activities of sternocleidomastoideus muscle and orbicularis oculi muscle, and cramp potential of sternocleidomastoideus muscle. DNA analysis confirmed HD mutation containing 49 CAG repeats. Genetic testing of his parents were both in normal range (his mother carried 18 /19 repeats and his father carried 16/19 repeats).

He was treated with valproic acid (25 mg bid), tiapride hydrochloride (150 mg tid), and artane (2 mg tid) during hospitalization and after discharge. The amplitude and frequency of tics reduced obviously and the tics in his hand diminished without obvious adverse effects. We follow up him regularly so far.

## Discussion and conclusion

Here we described a rare and interesting case of juvenile Huntington’s disease (JHD), a significant minority of HD commence before the age of 20 years, which presented tics as initial and prominent symptom. A review of the literature yielded initial symptoms and DNA analysis in 33 JHD patients when we used “juvenile Huntington’s disease”, “juvenile Huntington’s chorea”,“Huntington’s disease ”,“Huntington’s chorea” and “case report”, “clinical Study” for searching in PubMed, Embase, Cochrane Library, Web of Knowledge, CINAHL and ProQuest databases **(**Table 1 **)**. According to the results, unlike typically choreiform movement in adult-onset HD, the clinical manifestations of patients with JHD are atypical. The most prevalent symptoms are ataxia, followed by psychiatric manifestations, seizure, parkinsonism, dementia, dysphagia, dysarthria, and dystonia according to the previous reports **(**Table 1 **)**. Among them, two cases reported tics as initial and prominent manifestation in JHD, and only one with family history [3, 4]. Here we first reported a case mimic tics with low-range abnormal expansion of CAG without family history.

**Table 1:** clinical manifestation of JHD with DNA analysis and family history

patients	age at onset(year)	initial symptoms	Number of CAG repeats	Inheritance
this case	9	tics	47/19	no family history
1 [21]	6	character changes, anxiety, irritability, distractibility	unknown	paternal
2 [13]	2	rigid	250/19	maternal
3 [22]	17	balance and gait impairment	62/22	paternal
4 [22]	20	balance and gait impairment	61/22	paternal
5 [23]	6	seizure	115	paternal
6 [24]	2.5	hypokinetic/rigid syndrome	102	unknown
7 [25]	1	dystonia, speech impairment	256/14	paternal
8 [26]	10	depression	71	paternal
9 [27]	3	development delay, seizure	214	paternal
10 [28]	3.5	cognitive decline	84/15	maternal
11 [29]	3.5	speech impairment	108	maternal
12 [30]	3	ataxia	130–150/20	maternal
13 [31]	2	dysarthria, ataxia	53	paternal
14 [31]	4	dystonia	69	maternal
15 [31]	8	ataxia	41	paternal
16 [31]	13	visual hallucination	66	paternal
17 [32]	16	eating disorder	55/17	unknown
18 [33]	5	lethargy, poor balance	greater than 64	parental
19 [3]	4	excessive blinking	108/47	parental
20 [34]	5	ADHD	75	no family history
21 [35]	2	oral motor dysfunction and gait disturbance	160/60	maternal
22 [36]	5	behavioral disorders	52/15	maternal
23 [37]	6	seizure	140/20	parental
24 [38]	1.5	motor and speech delay	210–250/35	parental
25 [39]	3	speech impairment	95/17	no family history
26 [40]	20	seizure	60/21	parental
27 [41]	15	dystonia, parkinsonism	67/30	no family history, but father carried a reduced penetrance repeat
28 [42]	6	motor and speech regression	72	unknown
29 [4]	8	seizure	104/17	parental
30 [4]	10	tics	82/18	no family history
31 [4]	9	cognitive decline	76/17	parental
32 [4]	12	ataxia	74/17	parental
33 [43]	8	falls, ataxic gait and bradykinesia, seizure	82	no family history

*ADHD* attention-deficit/hyperactivity disorder

Tics are classified as idiopathic, namely TS, and secondary. The patient in this case had child-onset symptoms of tics, OCD, and ADHD, a typical presentation of TS [5]. However, both symptom of cervical dystonia and the abnormal EEG are rarely mentioned in TS. Thus, we screened for tics due to other disorders, such as head trauma, stroke, certain drugs, toxins, post-infection, neuroacanthocytosis, iron deposition, Wilson disease (WD), and HD. WD, iron deposition could be excluded by the absence of KF ring, normal iron and copper metabolism, normal structural MRI imaging. Head trauma, post infection and toxins were also excluded since the patient denied histories of streptococcus infection, trauma, and drug abuse. Normal structural MRI excluded stroke. Neuroacanthocytosis was excluded due to absence of hemolysis and normal blood smear. HD could not be excluded and it may be presented as tics, dystonia, seizure, behavior disorder similar in this patients. Mejia NI and colleagues have analyzed data on 155 patients with tics who did not fulfill the diagnostic criteria for TS, and found one patient diagnosed as HD by DNA analysis [6]. Several cases have report tics in HD, presented as blinking, face contraction, sniffing, jerk and multiple tics [3, 7–9]. Considering HD has various atypical symptoms including tics, especially for JHD, we made a DNA analysis of this boy and found abnormal CAG repeats. In contrast to most tics in HD patients, which had adult-onset [7–10], the initiated onset age in the present case was 9, a prevalent age at of onset for TS. Another possibility is the coexistence of both TS and HD in this patient. However, the typical progressive wax and wane disease course of TS was absent in this case, which gives less priority to the comorbidity [10]. In addition, genetic causes for TS are unknown, which makes it impossible to testify the hypothesis from genetic perspective. Both HD and tics are somewhat associated to a dopaminergic system dysfunction, but the molecular mechanism for the relationship between HD and TS need further investigated in future [11, 12].

We noticed that most JHD patients occur in families in which HD was already present, and paternal inheritance is more common, compared with maternal pattern from the literature review. However, the patient in the present case had negative family history, included by the negative finding in DNA analysis of the parents. A de novo occurrence of JHD in a family, reported by Post B, was confirmed the presence of a reduced penetrance repeat in the paternal side and the CAG expansion between generations [13]. Unlike the case mentioned above, the DNA analysis of both parents in this case were negative, thus the repeat expansion in this sporadic HD is likely to be explained by the mutation of normal HD gene.

From literature review, a large expansion is seen in most JHD, of which 29(88%) patients carried CAG repeats beyond 60, the largest reached 256. But CAG repeat of the present patient was below 60. Although longer CAG repeats predict earlier onset, CAG repeat length only accounts for about 60% of the variability [14]. The remaining are ascribed to additional genetic [15], environmental factors [14, 16] and other factors including somatic expansions in brain [17] or increasing in size [18].

The EEG in the patient in this case showed paroxysmal epileptic discharges during sleep without obvious seizure. Green JB and his colleagues have reported two JHD patients showing spikes in EEG without any type of seizure [19]. Positive spikes at 6–7 and 14 Hz could be found in 20% of healthy controls [20]. The underlying mechanism remains unknown. Since the abnormal spikes appear during sleep, the symptoms may also appear during sleep undetectably. The local spontaneous activities might account for cervical dystonia and intensive excitement of muscle in shorten position might result in cramp potential.

In summary, this case underlines marked phenotypic variability of HD, especially in JHD. HD should be considered in patients with juvenile-onset tic, especially with symptoms can’t be totally explained by TS, even in case of a negative family history although TS may be more prevalent. According to clinical phenomenology, we could not make the diagnosis of HD without genetic test. Therefore, molecular genetic test will became more helpful for the precision medicine of movement disorders in future. The further investigation should be focus on the relationship between TS and HD, especially at the level of pathophysiology and genetic etiology.

## Abbreviations

AAO: Age at onset
ADHD: Attention deficit hyperactivity disorder
AEEG: Ambulatory electroencephalography
DWI: Diffusion weighted imaging
HD: Huntington’s disease
JHD: Juvenile Huntington’s disease JHD
MRI: Magnetic resonance imaging
OCD: Obsessive-compulsive disorder
SWI: Susceptibility weighted imaging
TS: Tourette’s syndrome
